# HRBUST-LLPED: A Benchmark Dataset for Wearable Low-Light Pedestrian Detection

**DOI:** 10.3390/mi14122164

**Published:** 2023-11-28

**Authors:** Tianlin Li, Guanglu Sun, Linsen Yu, Kai Zhou

**Affiliations:** School of Computer Science and Technology, Harbin University of Science and Technology, No. 52 Xuefu Road, Nangang District, Harbin 150080, China; 2010400006@stu.hrbust.edu.cn (T.L.);

**Keywords:** low-light pedestrian detection dataset, lightweight model, wearable devices

## Abstract

Detecting pedestrians in low-light conditions is challenging, especially in the context of wearable platforms. Infrared cameras have been employed to enhance detection capabilities, whereas low-light cameras capture the more intricate features of pedestrians. With this in mind, we introduce a low-light pedestrian detection (called HRBUST-LLPED) dataset by capturing pedestrian data on campus using wearable low-light cameras. Most of the data were gathered under starlight-level illumination. Our dataset annotates 32,148 pedestrian instances in 4269 keyframes. The pedestrian density reaches high values with more than seven people per image. We provide four lightweight, low-light pedestrian detection models based on advanced YOLOv5 and YOLOv8. By training the models on public datasets and fine-tuning them on the HRBUST-LLPED dataset, our model obtained 69.90% in terms of AP@0.5:0.95 and 1.6 ms for the inference time. The experiments demonstrate that our research can assist in advancing pedestrian detection research by using low-light cameras in wearable devices.

## 1. Introduction

Over the past two decades, there has been a significant advancement in IoT and artificial intelligence technologies. As a result, researchers have turned their attention to developing intelligent wearable assistive systems that are made up of wearable cameras, sensors, computing components, and machine learning models [1]. This has led to an increase in studies aimed at assisting visually impaired individuals in various areas, such as travel [2], food [3], and screen detection [4]. Other areas of research include human-pet [5] or human-machine [6,7] interaction. Wearable devices combined with computer vision models are being used to help users observe things that are typically difficult to see. Despite the numerous studies on object detection using wearable devices, research on detecting humans in a scene is still limited, making it challenging to apply in areas such as nighttime surveillance, fire rescue, and forest inspections.

**Figure 1:**
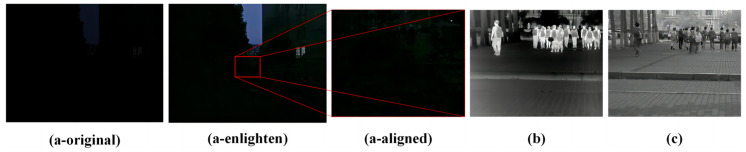
In starlight-level illumination environments, the imaging effects of visible light cameras, infrared cameras, and low-light cameras are as follows: (**a-original**) represents an image captured directly with a mobile phone; (**a-enlighten**) represents the image enhanced using the Zero DCE++ model; (**a-aligned**) represents the image in the enhanced version with the corresponding resolution. (**b**) represents an image captured with an infrared camera, and (**c**) illustrates an image captured with a low-light camera.

Since the maturity of convolutional neural networks in 2012, object detection algorithms have experienced vigorous development [8]. Single-stage object detection models represented by SSD [9] and YOLO [10], as well as two-stage object detection models depicted by Faster R-CNN [11] and FPN [12], have been proposed, achieving excellent results in terms of speed and accuracy. The maturity of object detection algorithms has also ushered in pedestrian detection algorithms into the era of deep learning. In order to fulfill the training data needs of machine learning and deep learning models, some usual pedestrian detection datasets have been proposed, like Caltech [13] and KITTI [14]. In recent years, datasets such as CityPersons [15], CrowdHuman [16], WIDER Pedestrian, WiderPerson [17], EuroCity [18], and TJU-Pedestrian [19] have been collected from cities, the countryside, and broader environments using vehicle-mounted cameras or surveillance cameras. These datasets enable the trained models to adapt to a broader range of scenarios. The EuroCity and TJU-Pedestrian datasets also include pedestrian data with low illumination conditions, aiming to achieve good recognition performance in terms of pedestrian detection models in nighttime scenarios. However, conventional cameras struggle to capture clear images under low-light conditions, significantly impacting data annotation and model recognition performance, as shown in Figure 1a.

Humans emit heat, which can be captured using infrared cameras in colder environments to distinguish pedestrians from the background, as shown in Figure 1b. As a result, OSU [20] proposed an infrared dataset collected during the daytime, and TNO [21] also provided a dataset that combines infrared and visible light captured at night. Later, with the development of research on autonomous driving, datasets such as CVC-14 [22], KAIST [23], and FLIR were introduced, which consist of pedestrian data captured using vehicle-mounted visible light-infrared cameras for modal alignment. Subsequently, the LLVIP dataset [24] was introduced to advance research on multi-spectral fusion and pedestrian detection under low-light conditions. Although infrared images can separate individuals from the background, they have a limited imaging distance and contain less detailed textures, making it difficult to distinguish pedestrians with high overlap.

Low-light cameras with CMOS chips specially designed to capture long-wavelength light waves can achieve precise imaging under starlight-level illumination conditions, as shown in Figure 1c. By considering the helpfulness of low-light images for pedestrian detection in low-light environments, we constructed the Low-Light Pedestrian Detection (HRBUST-LLPED, collected by Harbin University of Science and Technology) dataset in this study. The dataset consists of 150 videos captured under low-light conditions, from which 4269 keyframes were extracted and annotated with 32,148 pedestrians. In order to meet the requirements of wearable devices, we developed wearable low-light pedestrian detection models based on small and nano versions of YOLOv5 and YOLOv8. When considering the fact that the information captured by low-light cameras is relatively limited compared to visible-light cameras, we first trained the models on the KITTI, KAIST, LLVIP, and TJU-Pedestrian datasets separately and then fine-tuned them using our dataset. As a result, our trained models achieved satisfactory results in speed and accuracy.

Contributions. Our contributions cover several aspects.

(1) We have expanded the focus of pedestrian detection to low-light images and have constructed a low-light pedestrian detection dataset using a low-light camera. The dataset contains denser pedestrian instances compared to existing pedestrian detection datasets.
(2) We have provided lightweight, wearable, low-light pedestrian detection models based on the YOLOv5 and YOLOv8 frameworks, considering the lower computational power of wearable platforms when compared to GPUs. We have improved the model’s performance by modifying the activation layer and loss functions.
(3) We first pretrained our models on four visible light pedestrian detection datasets and then fine-tuned them on our constructed HRBUST-LLPED dataset. We achieved a performance of 69.90% in terms of AP@0.5:0.95 and an inference time of 1.6 ms per image.

In Section 2, we discuss the research progress on pedestrian detection datasets, object detection, and object detection using wearable devices. In Section 3, we present the details of our dataset. In Section 4, we describe the training methods and underlying architecture of our wearable low-light pedestrian detection model. In Section 5, we provide the experimental setup and evaluate the performance of our model. Finally, in Section 6, we conclude our work.

## 2. Related Work

This section will review some commonly used pedestrian detection datasets and then discuss some popular object detection models. Finally, we will introduce some research on applying object detection models to wearable devices.

### 2.1. Pedestrian Detection Datasets

The first dataset for pedestrian detection is MIT [25], which was proposed in 2000 and has images at a resolution of only 128×64. In the following years, the INRIA [26], Daimler [27], and TUD-Brussels [28] datasets were successively introduced, with increased image resolutions of 640×480 and varying numbers of images, ranging from hundreds to tens of thousands. Manually designed features characterized these earlier datasets.

However, with the development of neural networks and deep learning, early pedestrian detection datasets were too small to provide enough data for model fitting. In 2010, the Caltech [13] dataset was proposed, which consisted of 11 videos. The training set included six videos, with every third frame selected, while the test set comprised five videos, with every 30th frame chosen. Subsequently, in 2012, the KITTI [14] dataset was introduced, collected from onboard vehicle dashcams. The training set consisted of 7481 images, and the test set contained 7518 images, with a resolution of 1240×376. CityPersons [15], derived from the Cityscapes [29] dataset from 2017, comprised 2975 images in the training set, 500 images in the validation set, and 1575 images in the test set, with a resolution of 2048×1024. CrowdHuman [16], introduced in 2018, annotated complete pedestrians in the images and labeled the visible parts and heads of each pedestrian. WIDER Pedestrian, a 2019 dataset, focused on vehicle and pedestrian detection and drew images primarily from roadside surveillance and onboard cameras. WiderPerson [17], proposed in 2019, concentrated on pedestrian detection in outdoor scenes rather than traffic scenarios, with the data mainly collected from web sources. The EuroCity [18] dataset, introduced in 2019, captured pedestrian data under various lighting conditions, including daytime and nighttime, from multiple cities in Europe, making it the first all-weather pedestrian detection dataset. TJU-Pedestrian [19], a pedestrian detection dataset proposed by Tianjin University in 2020, covers two main scenarios: road traffic and the campus and consists of pedestrian data from both day and night. It is currently one of the most comprehensive pedestrian detection datasets available.

All the pedestrian detection datasets mentioned above were captured using visible light cameras. However, capturing pedestrians clearly in low-light scenarios is challenging using conventional visible light cameras. In 2015, the KAIST dataset [23] introduced the first pedestrian detection dataset that includes both infrared and visible light modalities, taking into account the illumination variations between day and night that can affect the performance of automated systems. The KAIST dataset primarily provides 12 pairs of fully aligned visible light and infrared videos, with six pairs used for training and six pairs for testing. The CVC-14 dataset [22], proposed in 2016, is a dataset that simultaneously collects visible light and infrared images, primarily targeting autonomous driving scenarios. It consists of 7085 training images and 1433 test images with a resolution of 640×512. One of the drawbacks of this dataset is the lack of complete alignment between the visible light and infrared modalities. The FLIR Thermal dataset, introduced in 2018, mainly contains 9711 infrared images and 9233 RGB images for model training and validation. It aims to promote the use of FLIR’s infrared cameras in autonomous driving systems. The LLVIP dataset [24], proposed in 2022, constructs a dataset that corresponds to visible light and infrared images. It is primarily used for image fusion, pedestrian detection, and modality transfer tasks.

### 2.2. Object Detection

The development of object detection models is based on the success of AlexNet and VGGNet. Early object detection models can be divided into two categories: two-stage models and one-stage models.

The pioneering work of the two-stage models is the R-CNN [30] model in 2014, which is also the first deep learning-based object detection model. The main idea of the two-stage object detection model is to select rough regions of interest that may contain objects after extracting image features through convolution and then classify specific objects from the aligned regions of interest. Subsequently, Fast R-CNN [31] and Faster R-CNN [11] improve the object selection process, reducing computational costs and enabling end-to-end training of the entire network. In order to better detect multi-scale objects, MS-CNN [32] and FPN [12] were introduced into two-stage object detection algorithms, improving their accuracy and adaptability to complex scenes.

One-stage object detection models directly divide extracted feature maps into N× N regions to predict objects for each region, eliminating the need to predict regions of interest and improving the speed of object detection models. Popular models in this category are the YOLO [10] and SSD [9], and YOLO has evolved from YOLOv1 to YOLOv8. Although YOLOv1 [10] reduces computational complexity by directly regressing the position of the bounding box, it is not effective in detecting dense and small objects. YOLOv2 [33] and YOLOv3 [34] improved on this basis and combined the PassThrough method, anchor point, and FPN to predict multi-scale targets. YOLOv4 [35] and YOLOv5 [36] further improve performance by integrating the CSP and SPP structures, adaptive anchor calculation, and Focus operations. YOLOv6 [37] and YOLOv7 [38] introduced RepVGG and ELAN modules to improve performance, respectively, whereas YOLOv8 improved the CSP structure, prediction head, and loss function to further improve calculation speed and accuracy.

### 2.3. Object Detection on Wearable Devices

Research on object detection models for wearable devices can be roughly divided into two categories based on the data source: intelligent assistive devices based on everyday life images and assistive devices based on AR platforms.

In AR-based research, Eckert et al. [39] combined HoloLens with YOLOv2 to design an assistive system that provides audio navigation for the visually impaired. Bahri et al. [40] deployed and evaluated YOLOv3 and Faster R-CNN on the HoloLens platform. Park et al. [41] used the Mask R-CNN model to achieve object segmentation in AR devices and evaluated its application in matching, inspecting, and maintaining items in intelligent manufacturing. Park et al. [6] employed RetinaNet for object detection and used AR technology as a platform to provide intelligent assistance for collaborative robots. Dimitropoulos et al. [7] utilized AR and deep learning for wearable recognition, enabling interaction between human operators and robots through voice or gestures.

In the research based on everyday life images, Han et al. [3] used Faster R-CNN for food object detection, implemented it in glasses as a wearable device, and automatically captured images of dining scenes while estimating the number of chewing motions. Kim et al. [5] utilized wearable devices to recognize and predict dog behavior and evaluated the performance of Faster R-CNN and YOLOv3/v4. Li et al. [4] employed wearable sensors and the YOLOv5 model to detect electronic screens, evaluating the duration of electronic screen usage. Arifando et al. [42] proposed a lightweight and high-precision bus detection model based on YOLOv5 to assist visually impaired individuals in boarding the correct bus and getting off at the right bus stop. Maya-Martínez et al. [43] introduced a pedestrian detection architecture based on Tiny YOLOv3, implemented in low-cost wearable devices as an alternative solution for assisting visually impaired individuals. Although these studies provide a good basis for pedestrian detection using wearable devices, they do not consider pedestrian detection at night in low light.

## 3. The HRBUST-LLEPD Dataset

This section will first provide a detailed introduction to the proposed Low-Light Pedestrian Detection (HRBUST-LLPED) dataset, including how we capture the visual data, preprocess and select the keyframes, and annotate the pedestrians. Then, we will briefly compare our constructed dataset with other commonly used datasets and analyze the advantages and disadvantages of the HRBUST-LLPED dataset.

### 3.1. Dataset Build

Data Capture: The low-light camera we used is the Iraytek PF6L, with an output resolution of 720×576/8μm, a focal length of F25mm/F1.4, and a theoretical illuminance resolution of 0.002 Lux. The camera is attached to a helmet. We wear the helmet to capture data to simulate the real perspective of humans. We mainly shoot campus scenes from winter to summer. The collection time in winter was 18:00–22:00, and in summer, it was between 20:00 and 22:00. In total, we collected 150 videos with a frame rate of 60 Hz. The length of the videos ranged from 33 s to 7 min and 45 s, with an average length of 95 s and a total of 856,183 frames.

Data Process: First of all, when considering the thermal stability and high sensitivity of CCD (CMOS) in the video acquisition process of low-light cameras, noise will inevitably be introduced into the video. Additionally, since the frame rate of the video is 60 Hz and the difference in pedestrian poses between adjacent frames is minimal, we first use a smoothing denoising technique with the neighboring two frames to enhance the current frame. Next, when considering that the pedestrian gaits in the video are usually slow, there is significant redundancy in the pedestrian poses. Therefore, we select one frame every 180 frames (i.e., 3 s per frame) as a keyframe. We then remove frames that do not contain pedestrian targets and search for clear frames within a range of ±7 frames of the blurred frames to update the keyframes. Ultimately, we obtained 4269 frames as the image data for constructing our low-light pedestrian detection dataset.

Data Annotation: We used the labelImg tool to annotate the processed image data manually. For each person present in the image with less than 90% occlusion (i.e., except for cases where only a tiny portion of the lower leg or arm is visible), we labeled them as “Pedestrian”. We cross-referenced the uncertain pedestrian annotations with the original videos to avoid missing pedestrians due to visual reasons or mistakenly labeling trees as pedestrians. As a result, we obtained a total of 32,148 pedestrian labels.

### 3.2. Dataset Analysis

In this section, we will analyze the performance of our dataset and compare it to other datasets. We will evaluate the strengths and limitations of our dataset.

Analyze HRBUST-LLPED itself

**Figure 2:**
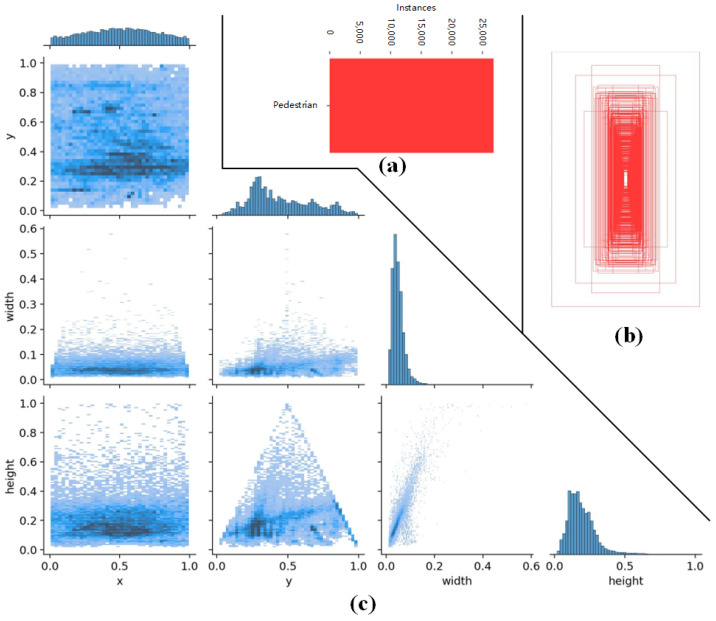
The distribution of the HRBUST-LLEPD training set. (**a**) represents the distribution of pedestrian instances in the dataset. (**b**) illustrates the sizes and quantities of the bounding boxes. (**c**) shows the distribution of the bounding box in the training set.

Our dataset consists of 4269 images annotated with 32,148 pedestrians. We evenly divided the dataset into training and testing sets with a 5:1 image ratio. Specifically, we took five consecutive images for training and one for testing. As a result, the constructed training set contains 3558 images with annotations for 26,774 pedestrians, and the testing set contains 711 images with annotations for 5374 pedestrians. Since both the training and testing sets were uniformly selected, we primarily focus on analyzing the training set, as shown in Figure 2. Figure 2a displays the distribution of pedestrian instances in the dataset, whereas Figure 2b illustrates the sizes and quantities of the bounding boxes. Figure 2c demonstrates the relationships between the center point’s x-co-ordinate, y-co-ordinate, width, and the height of the bounding boxes. From the figure, we observe that the pedestrian boxes are distributed throughout the entire image. The distribution of the x-co-ordinate of the center point is relatively uniform, while the y-co-ordinate is more concentrated in the middle and lower parts of the image. The width mainly ranges from 5% to 20% of the image, and the height mainly ranges from 10% to 40% of the image. These characteristics align with the nature of pedestrian data, indicating that the dataset covers a wide range of pedestrian positions.

Compare with other datasets

Compared with other pedestrian detection datasets. We used the processed image dataset instead of the raw video as the training data, and when training the model from scratch using only our dataset, this results in low accuracy and poor robustness. Therefore, we extracted pedestrian labels and images containing only pedestrian labels from commonly used KITTI, TJU-PED, LLVIP, and KAIST datasets to build dedicated pretrained pedestrian detection datasets. The details of each processed dataset are presented in Table 1. For the specific handling of publicly available datasets, we applied the following procedures:

KITTI: This dataset contains nine categories, including Pedestrian, Truck, Car, Cyclist, DontCare, Misc, Van, Tram, and Person_sitting. We retained only the Pedestrian labels and extracted the corresponding training set images based on those labels.

TJU-PED: The original dataset of TJU-PED is TJU-DHD, which includes two scenes, “Traffic” and “Campus”. The Traffic scene has five labels: Pedestrian, Rider, Car, Van, and Truck. The Campus scene consists of two labels: Pedestrian and Rider. As our research focuses on pedestrians, and there are differences between riders and pedestrians in terms of appearance, we retained only the Pedestrian labels and their corresponding images from both scenes, resulting in the TJU-PED-Traffic and TJU-PED-Campus datasets. Finally, we merged the datasets to obtain the TJU-PED dataset.

LLVIP: This dataset includes infrared and visible light modalities. Due to the significant differences between the infrared and visible light modalities, we only used the visible light modality for pretraining, resulting in the LLVIP-PED dataset.

KAIST: This dataset provides 12 pairs of visible-infrared videos. For the visible light videos, we selected every third frame as a key frame from the training set, retaining the images with pedestrian labels. We selected one image from the testing set every 30 frames, retaining the images with pedestrian labels. This process yielded the KAIST-PED dataset.

**Figure 3:**
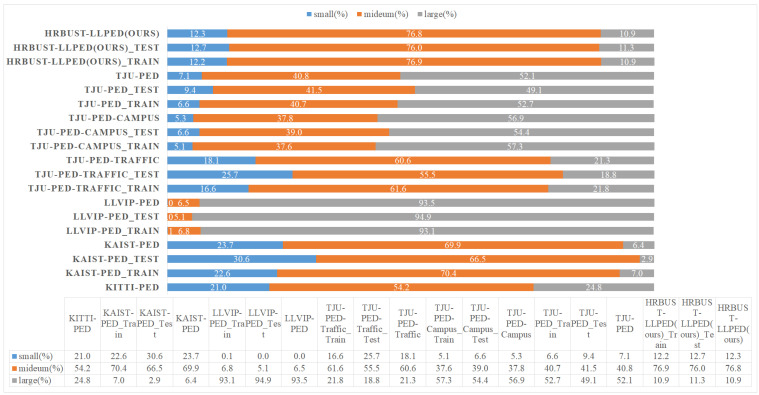
The distribution of pedestrian size in different datasets.

Object Size: We compared the distribution of objects of different sizes in HRBUST-LLPED and several other datasets. We defined the object sizes following the same criteria as the MS COCO dataset. Objects with an area smaller than 32×32 square pixels were classified as small objects, objects with an area ranging from 32×32 to 96×96 square pixels were classified as medium-sized objects, and objects with an area larger than 96×96 square pixels were classified as large objects. The specific distribution of object sizes in the dataset is shown in Figure 3. It can be observed that the HRBUST-LLPED dataset covers large, medium, and small objects, with a focus on medium-sized objects. Notably, in the HRBUST-LLPED dataset, the distribution of objects of different sizes is consistent between the training and testing sets. We consider this consistency to be beneficial for training the models.

**Figure 4:**
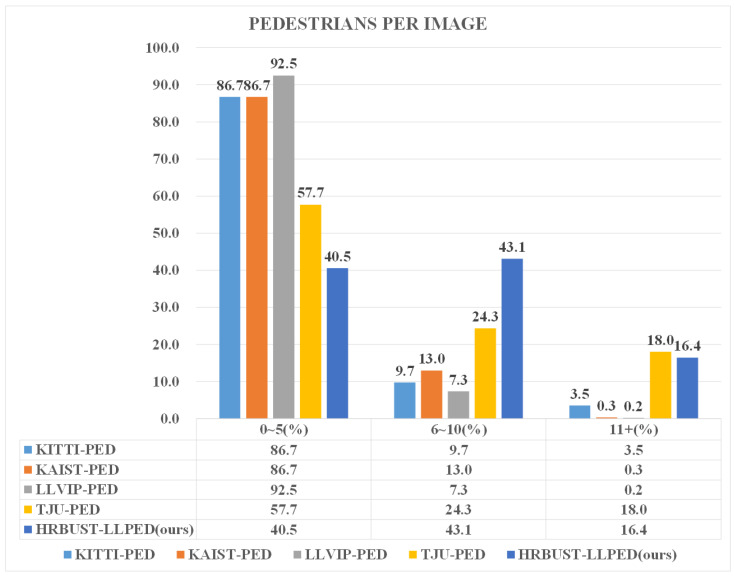
The distribution of pedestrian numbers per image in different datasets.

Pedestrian Density: Pedestrian density is a crucial factor influencing the performance of pedestrian detection models. High pedestrian density implies many pedestrians overlapping with each other, and excessive occlusion can impact the model’s ability to recognize pedestrians. Therefore, we evaluated the density of pedestrians in different datasets. Specifically, we counted the number of pedestrians in each image based on the dataset’s annotations. We categorized the images into three groups: images with 0 to 5 pedestrians, images with 6 to 10 pedestrians, and images with 11 or more pedestrians. The results are presented in Figure 4. From the figure, we can observe that in the HRBUST-LLPED dataset, images with 6 to 10 pedestrians have the highest proportion, and there is also a significant proportion of images with 11 or more pedestrians. When combining Figure 4 with Table 1, it can be noticed that the HRBUST-LLPED dataset has a relatively high number of pedestrians, with the highest mean pedestrians per image. The distribution of pedestrian numbers is also relatively uniform.

Therefore, when compared to the other datasets, the HRBUST-LLEPD dataset has the following advantages:

- HRBUST-LLPED is a pedestrian detection dataset for low-light conditions (starlight-level illumination). It captures clear images using low-light cameras, making it suitable for developing pedestrian detection algorithms in low-light environments.
- The dataset contains abundant pedestrian annotations, covering pedestrians of various sizes. Each image includes a substantial number of pedestrians, with significant occlusion between the pedestrians and between the pedestrians and the background. This enables comprehensive training and evaluation of the model’s pedestrian recognition capability.
- The dataset captures scenes from different seasons, ranging from winter to summer, and includes weather conditions such as snow, sunny, and cloudy. This diversity in weather conditions ensures the model’s robustness across different weather scenarios.

Nevertheless, our constructed dataset has a limitation due to the constraints of the data collection equipment, resulting in a relatively low resolution. Therefore, this dataset may not be suitable for training models with very deep architectures from scratch, as those models might struggle to fit the data fully.

## 4. Wearable Low-Light Pedestrian Detection

**Figure 5:**
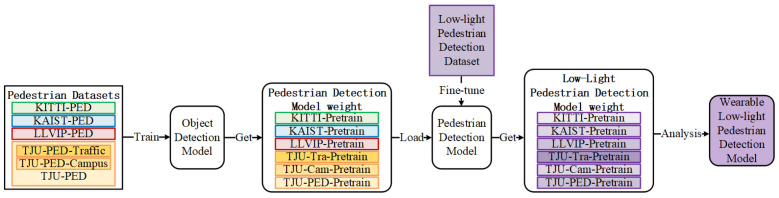
The training framework of the proposed model.

The training framework of this paper is illustrated in Figure 5. We first utilized the processed visible light pedestrian detection datasets from Section 3, namely, KITTI-PED, KAIST-PED, LLVIP-PED, TJU-PED, and their subsets, to train and test the pedestrian detection models. Subsequently, the trained wearable pedestrian detection models were transferred to our HRBUST-LLPED dataset for further training, resulting in wearable low-light pedestrian detection models with varying performance. Finally, we compared and analyzed the performances of the models to select high-accuracy and fast-speed models.

**Figure 6:**
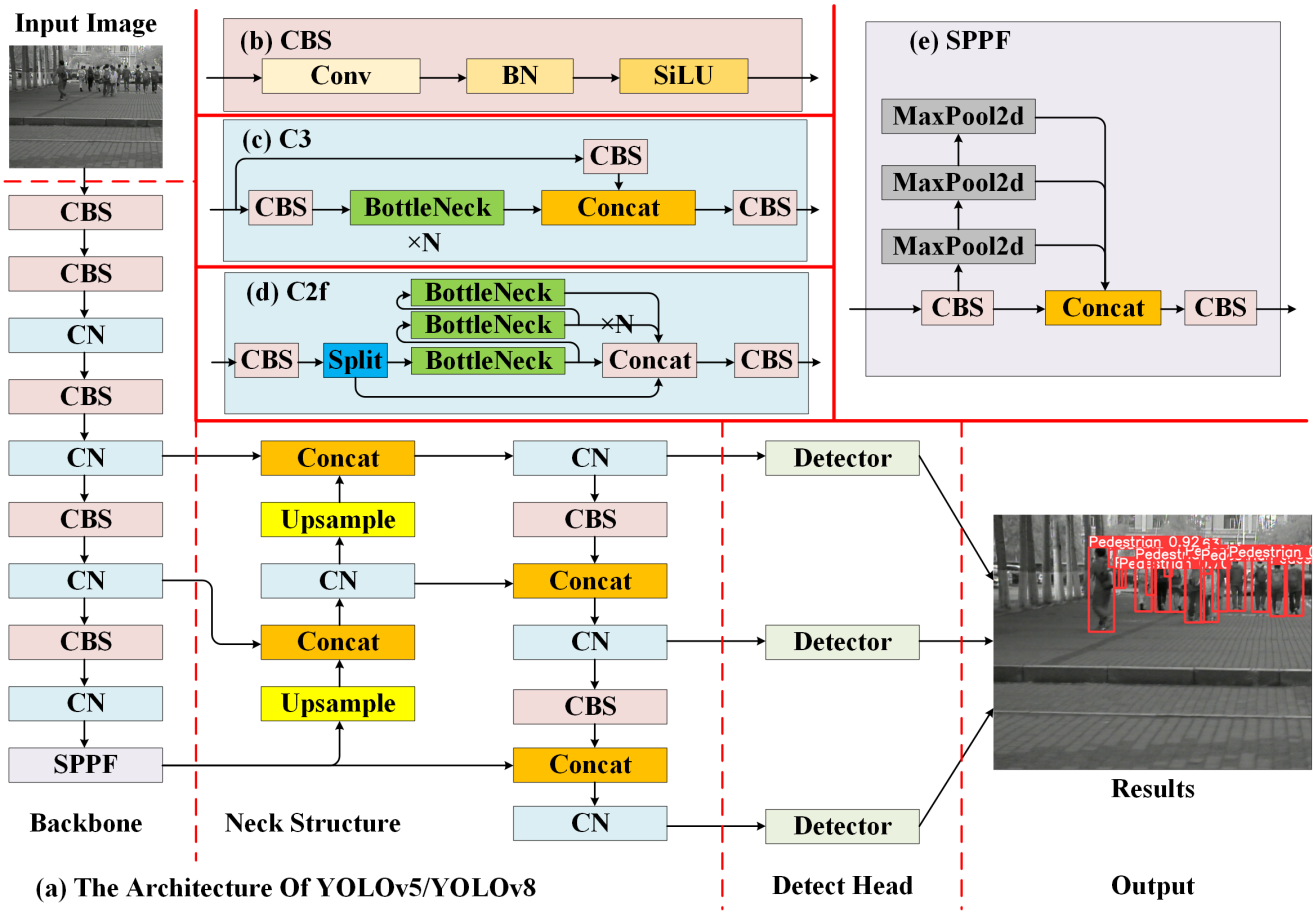
The architecture of YOLOv5/YOLOv8.

YOLOv5 and YOLOv8 are single-stage object detection models developed by the Ultralytics team. They are well-known for their high integration, fast deployment, and applicability to various downstream tasks. One significant characteristic of these two models is that they consist of five different model versions, ranging from small to extra large, denoted as nano (n), small (s), middle (m), large (l), and extra large (x), based on the depth and width of the models. These five model versions allow researchers to choose the depth and width of the model according to the specific requirements of their application scenarios. In this study, our application scenario focuses on wearable pedestrian detection in low-light conditions at night. Therefore, we select the nano and small versions as baselines for modification and experimentation. The overall structures of these two models are similar, as shown in Figure 6a, consisting of three components: backbone, neck structure, and detect head.

Backbone: Its primary function is to extract features from input images, primarily including CBS blocks, CN blocks, and SPPF blocks. The CBS block consists of a convolutional block, a BN layer, and a SiLU activation layer, as shown in Figure 6b. For the input feature *x*, the computation output of the SiLU activation function is given by Equation (1).

(1) $$\mathrm{SiLU}\left(x\right)=x*\mathrm{sigmoid}\left(x\right)=\frac{x}{1+e^{-x}}$$

In order to implement the CN block, YOLOv5 utilizes the C3 block, which consists of three CBS blocks and n concatenated BottleNeck blocks, as shown in Figure 6c. YOLOv8, on the other hand, employs the C2f block, composed of three CBS blocks and n concatenated BottleNeck blocks, as illustrated in Figure 6d. The structure of the SPPF block is depicted in Figure 6e and includes two CSB blocks, three max-pooling layers, and a concatenation operation. In this study, to accelerate the detection speed of the model, the SiLU activation function is replaced with a more straightforward ReLU activation function.

Neck Structure: Its main function is to fuse the multi-scale features extracted from the backbone network, thereby interacting with semantic information of various granularities, drawing inspiration from the ideas of feature pyramids and pixel aggregation networks. In this section, besides implementing the CN block using their respective methods, YOLOv5 adds a 1×1 CBS block before each upsampling layer.

**Figure 7:**
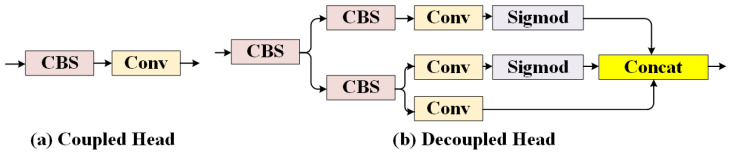
The architecture of different Detect Heads.

Detect Head: Its main function is to generate the detection results of the model based on the interacted feature information. In this aspect, YOLOv5 utilizes the Coupled head, which directly performs classification using a convolutional layer, as shown in Figure 7a. On the other hand, YOLOv8 employs the Decoupled head, as illustrated in Figure 7b, which separates the processes of classification and regression to improve the convergence speed and accuracy of the model.

Training: During the training process of the object detection model, the main focus is on training the model’s ability to correctly classify the target’s category and accurately regress its position. In the task of low-light pedestrian detection, when training the model’s classification ability, the objective is to correctly classify pedestrians from the background, thereby utilizing pedestrian samples more effectively for model training supervision. Therefore, we employ Varifocal Loss to train the model’s classification ability, which is calculated as follows:

(2) $$\mathrm{VFL}\left(p,q\right)=\left\{\begin{array}{cc} -q(q\log(p)+(1-q)\log(1-p)) & q>0\\ -\alpha p^{\gamma }\log\left(1-p\right) & q=0 \end{array}\right.$$

where p is the probability that the model predicts that the target is a pedestrian, and *q* is the intersection over union (IoU) of the predicted bounding box and ground truth.

During the training process of regressing the target bounding boxes, we aim to make the predicted boxes as close as possible to the ground truth boxes. In other words, we aim to maximize the IoU between the predicted and ground truth boxes, minimize the distance between their center points, and ensure consistency in the aspect ratio. In order to achieve this, we utilize CIoU Loss to train the model’s regression capability, which is calculated as follows:

(3) $$L_{CIoU}=1-IoU+\frac{\rho^{2}\left(b,b^{gt}\right)}{c^{2}}+\alpha v$$

where ρ represents the Euclidean distance, b and $b^{gt}$ denote the predicted and ground truth center points, *c* is the diagonal distance of the minimum bounding box, α is a balancing parameter calculated as in Equation (4), and *v* represents the aspect ratio similarity, computed according to Equation (5).

(4) $$\alpha =\frac{v}{(1-IoU)+v}$$

(5) $$v=\frac{4}{\pi^{2}}{\left(\arctan\frac{w^{gt}}{h^{gt}}-\arctan\frac{w}{h}\right)}^{2}$$

Therefore, the formula of the Loss function *L* of our model training is as follows:

(6) $$L=L_{VFL}+L_{CIoU}$$

## 5. Experiments

In this section, we utilize the low-light pedestrian detection dataset we constructed and publicly available data to train the pedestrian detection models described in Section 4. We then evaluate the accuracy and speed of the models. We aim to train a baseline model as a foundation for future research.

### 5.1. Evaluate Metric

Similar to the well-known object detection dataset MS COCO, we evaluate the performance of our models on pedestrian detection using precision (P), recall (R), AP@0.5, and AP@0.5:0.95. The calculation methods for the evaluation metrics in this paper are as follows:

True Positive (TP): The prediction is positive, and it is correct.

False Positive (FP): The prediction is positive, but it is wrong.

False Negative (FN): The prediction is negative, but it is wrong.

Precious (P): Describe the proportion of TP in the detection results, calculated as follows: Precious $=\frac{TP}{TP+FP}$.

Recall (R): Describe the detection rate of labeled pedestrians, calculated as follows: Recall $=\frac{TP}{TP+FN}=\frac{TP}{GT}$.

AP@0.5 (AP50): When the IoU threshold is set to 0.5, the area enclosed by the PR curve and the co-ordinate axis is calculated, i.e., $AP=\int_{0}^{1}P\left(r\right)dr$.

AP@0.5:0.95 (AP): Similar to the MS COCO dataset, using 0.05 as the step for changing the IoU threshold, multiple APs are obtained by changing the IoU from 0.5 to 0.95, and then the average value is taken.

Missing Rate (MR): $=\frac{FN}{GT}$.

False Detection Rate (FDR): $=\frac{FP}{TP+FP}$.

Incorrect Localization Rate (ILR): The proportion of prediction boxes with an IoU threshold between 0.2 and 0.5 with ground truth boxes.

### 5.2. Implementation Details

The wearable low-light pedestrian detection models in this paper were implemented based on YOLOv5 and YOLOv8, with activation and loss function modifications to adjust training performance. We used four datasets: KITTI, TJU-PED, KAIST, and LLVIP. We loaded the pretrained weights of the general object detection dataset MS COCO to train our models. The training was conducted for 50 epochs with an initial learning rate of 0.001, which is reduced to 0.0005 after 20 epochs. For the KAIST dataset, we trained the model with an input resolution of 640×640 and set the batch size to 64. For the KITTI, TJU-PED, and LLVIP datasets, we trained the model with input resolutions of 640×640 and 1280×1280 and set the batch size to 64 and 16, respectively. Subsequently, when transferring the pretrained model to our dataset, we trained it for 50 epochs, with an initial learning rate of 0.001, which was reduced to 0.0005 after 20 epochs. The input resolution was 640×640, and the batch size was 64. We used two devices for training and testing the models. Both devices have an 11th Gen Intel Core i7-11800H @ 2.30 GHz CPU and 32 GB of memory. One device has an NVIDIA GeForce RTX 3080 Laptop GPU at 16 GB, whereas the other has two NVIDIA GeForce RTX 2080Ti GPUs at 11 GB.

### 5.3. Experimental Results

We first trained wearable pedestrian detection models based on YOLOv5s, YOLOv5n, YOLOv8s, and YOLOv8n directly on the HRBUST-LLPED dataset we constructed. In the subsequent text and tables, these four basic pedestrian detection models are abbreviated as YOLOv5s, YOLOv5n, YOLOv8s, and YOLOv8n, and the original YOLO model is marked with a “-o” suffix at the end (i.e., YOLOv5s-o, YOLOv5n-o, YOLOv8s-o, and YOLOv8n-o). The experimental setup was consistent with the experiment using the KAIST dataset for pretraining. The training results are shown in Table 2. The table shows that the YOLOv5s-o model achieves the best performance in terms of precious. The YOLOv5s model achieves the best performance in terms of recall rate and AP@0.5. On the other hand, the YOLOv8s model performs best in terms of AP@0.5:0.95. In summary, the YOLOv5s model can quickly fit the approximate distribution of our dataset, whereas the YOLOv8s model can more accurately identify the specific locations of pedestrians in the scene. Furthermore, the YOLOv8n model has the fastest inference speed, reaching 1.6 ms. From Table 2 and Table 3, we can observe that our improved pedestrian detection models have a 1–2% improvement in terms of AP and 0.3–0.9 ms improvement in terms of inference time.

Subsequently, we trained the models on four visible light pedestrian detection datasets (KITTI-PED, KAIST-PED, LLVIP-PED, and TJU-PED) and evaluated their performance on our constructed HRBUST-LLPED dataset. The experimental results are presented in Table 3, Table 4, Table 5, Table 6, Table 7 and Table 8. Each table includes the model’s name (column 1), the input resolution of the model during training (column 2), the results of the model trained and tested on the visible light pedestrian detection dataset (columns 3 to 6), the test results after transferring the model to the HRBUST-LLPED dataset (column 7 to 10), and the model’s inference time (column 11). The inference time was measured on an NVIDIA GeForce RTX 3080 Laptop GPU with 16 GB of memory. The bold numbers indicate the optimal values for each evaluation metric. From Table 3, Table 4, Table 5, Table 6, Table 7 and Table 8, it can be observed that our dataset is more difficult than the KITTI dataset. For the KAIST and LLVIP datasets, the pedestrians captured in the nighttime images are blurred and submerged in the background, making it more difficult to detect. When compared to the TJU series of datasets, our dataset still has a gap in terms of resolution diversity and data quantity, which is one of the issues we will address in our future work.

From Table 4 and Table 5, it can be observed that pretraining on the KITTI-PED and KAIST-PED datasets leads to approximately a 1% improvement in accuracy, a 4–7% improvement in recall rate, a 3% improvement in AP@0.5, and a 5–7% improvement in AP for all four models. However, there is no improvement in inference speed. Training the models with an input resolution of 1280×1280 on the KITTI-PED dataset effectively improves model performance. On the other hand, when transferring the models to the HRBUST-LLPED dataset, it seems more beneficial to train them at a resolution of 640×640, as it improves the accuracy of the transferred models. This phenomenon corresponds to the resolution of the images in the dataset. Additionally, it is counterintuitive that changing the input resolution does not affect the inference speed of the YOLOv5 models, whereas it significantly slows down the YOLOv8 models by nearly four times.

As shown in Table 3, for the YOLOv5s model, features with a resolution of 640×640 are more effective for pedestrian recognition when trained on the LLVIP-PED dataset. This observation is supported by Figure 3, which indicates that the LLVIP-PED dataset contains significant annotations for large-scale pedestrians. Therefore, it seems that the YOLOv5s model is less sensitive to larger-sized objects.

Table 6, Table 7 and Table 8 further confirm the aforementioned conjecture, and we can draw the following preliminary conclusions:

- The model with the highest detection of precious is based on YOLOv5s pretrained on the TJU-PED dataset, with an accuracy of 95.15%. The model with the highest recall rate and AP@0.5 is based on YOLOv8s pretrained on the TJU-PED-Campus dataset, with 91.66% in terms of recall and 96.34% in terms of AP@0.5. The models with the highest AP@0.5:0.95 are based on YOLOv8s pretrained on the TJU-PED dataset, achieving a value of 69.90%. The fastest model is based on YOLOv8n, with an inference speed of 1.6 ms per image.
- Among the four selected models, YOLOv8s performs the best, achieving approximately a 3% higher for AP@0.5:0.95 than YOLOv5s. YOLOv8n and YOLOv5s have similar accuracies, but YOLOv8n is approximately 1.5 ms faster.
- For most pretrained datasets, training the model with pedestrian sizes closer to the target dataset leads to better performance when transferring the model.
- For the YOLOv5 models, the input image resolution does not affect the inference speed, whereas for the YOLOv8 models, the input image resolution impacts the model’s speed.

### 5.4. Further Analysis

After obtaining the training results in Section 5.3, we will further analyze the impact of different pretrained datasets on transferring the models to the HRBUST-LLPED dataset, as well as the performance of our trained models during actual predictions in this section.

**The impact of different pretrained datasets on transferring the models to the HRBUST-LLPED dataset.**

**Figure 8:**
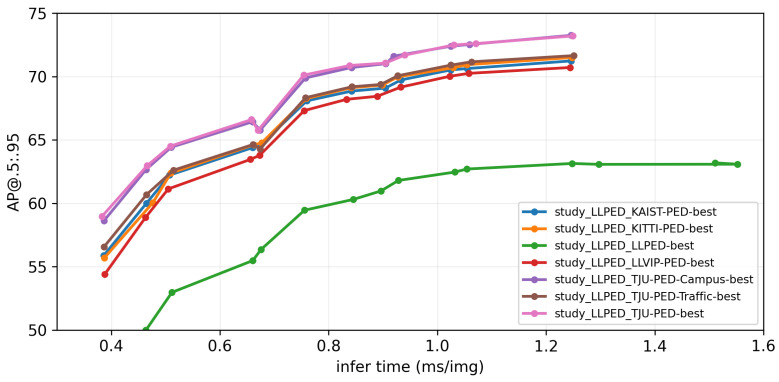
Model performance when trained on different datasets.

In order to investigate this issue, we extracted the model weights with the highest AP@0.5:0.95 metric on each pretrained dataset, labeled as “best” for that dataset. We then set the input image resolution, starting from 256×256 square pixels, and increased it by 32 in stride, iterating up to 1280×1280 square pixels. We tested the models with different pixel inputs to assess their recognition speed and accuracy for different image sizes. All tests were conducted on an NVIDIA GeForce RTX 3080 Laptop GPU with 16GB of memory. The final results are shown in Figure 8, where each line is labeled in the format “study_Test Dataset_Train Dataset-best”. First, it can be observed that training the models with other datasets before the target dataset improves the model’s performance to some extent. Second, it can be seen that TJU-PED-Campus and TJU-PED are the most effective datasets for model training. This is likely because our dataset and these two datasets primarily focus on pedestrians within campus environments, making their distributions similar and facilitating model transfer. Additionally, LLVIP has the most negligible impact on model training, possibly because the LLVIP dataset contains many large objects that do not align well with the distribution of the HRBUST-LLPED dataset, requiring more time to train the model.

**The performance of our trained models during actual predictions.**

**Figure 9:**
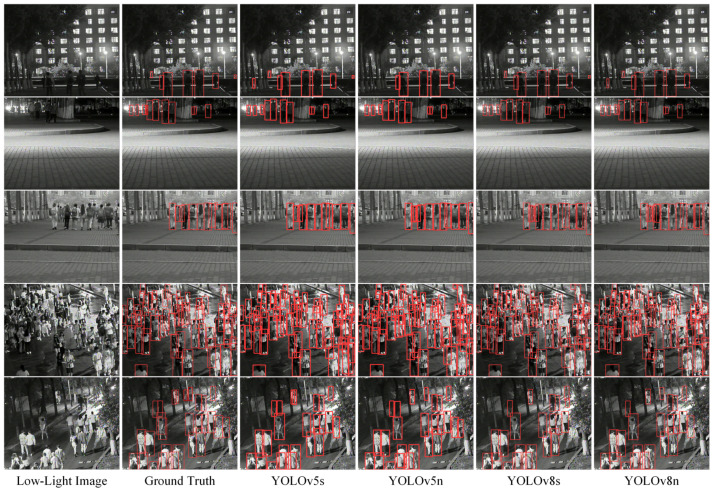
The qualitative result of different models on the HRBUST-LLPED dataset.

We have visualized several examples and their detection results to demonstrate each model’s effectiveness in Figure 9. In the figure, from top to bottom, there are five test images, and from left to right, these show the original low-light image, ground truth labels, and predicted labels from YOLOv5s, YOLOv5n, YOLOv8s, and YOLOv8n, respectively. From the figure, it can be observed that all four wearable low-light pedestrian detection models can generally detect pedestrians present in the scenes. Additionally, the YOLOv5s-based and YOLOv8s-based wearable low-light pedestrian detection models can detect partially occluded pedestrians, such as the pedestrian in the far right of the first row, where only the upper body is visible. However, these four models still have room for improvement in detecting pedestrians with high overlap, as seen in the left-middle part of the last row, where all four models identify two overlapping individuals as single persons.

**The quantitative and qualitative analysis of the failure cases.**

In order to further analyze the characteristics of the HRBUST-LLPED dataset, we chose the models that pretrained on the TJU-PED dataset and were transferred to our dataset. In order to make the analysis results more accurate, we set the confidence threshold for detecting pedestrians to 0.3. We evaluated the model using three indicators: the missing rate, false detection rate, and incorrect localization rate. The smaller these indicators, the better the model. The experimental results are shown in Table 9. From the table, it can be found that the models with a pretrained resolution of 1280×1280 perform better. In addition, the YOLOv5s model has the lowest false negative rate, the YOLOv8n model has the lowest false positive rate, and the YOLOv8s model has more accurate localization.

**Figure 10:**
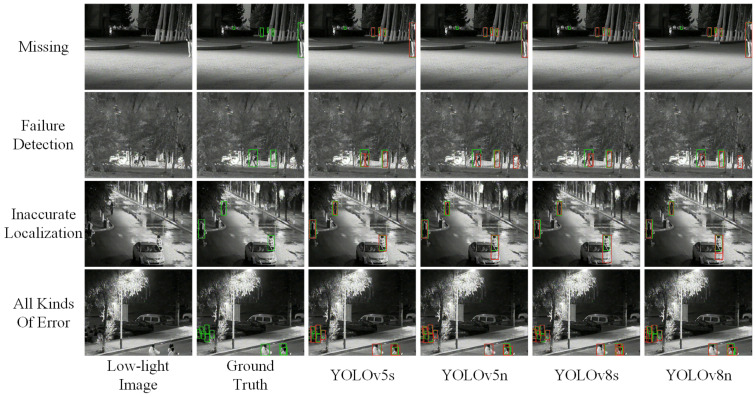
The cases of different kinds of failure.

Subsequently, we selected the models with a pretrained resolution of 1280×1280 for qualitative analysis, as shown in Figure 10. The green bounding box indicates the ground truth, while the red indicates that predicted by the model. From the first row, it can be found that pedestrians in dark places with only a small part of the area exposed due to severe occlusion are prone to miss detection. From the second line, it can be found that areas with overexposure and severe occlusion are more prone to false detection. From the third line, it can be found that people who are obscured are more likely to be in an inaccurate location. Some pictures may cause multiple errors, as shown in the fourth line. Overall, our dataset contains occluded and poorly exposed images, posing challenges to pedestrian detection models.

## 6. Conclusions

In this paper, we have constructed a low-light pedestrian detection dataset specifically targeting campus scenes. By using low-light cameras to capture clear images during nighttime, our dataset is suitable for developing pedestrian detection algorithms in low-light environments. We have also provided wearable low-light pedestrian detection models based on the well-known YOLO architecture to test the validity of the HRBUST-LLEPD dataset. We pretrained the models on the public pedestrian datasets and fine-tuned them on our dataset. The experimental results provide a baseline performance for low-light pedestrian detection research. We hope this study will contribute to developing wearable nighttime pedestrian assistance systems.

## Figures and Tables

**Table 1:** Comparison of pedestrian data between HRBUST-LLPED and the other datasets.

	Num. of Train Images	Num. of Test Images	Num. of Train Instances	Num. of Test Instances	Resolution	Image Type	Day/ Night	Pedestrians per Image
KITTI-PED	1796	-	4708	-	1238 × 374	Visible	Day	2.62
KAIST-PED	7595	1383	24,304	4163	640 × 512	Visible	Day	3.17
LLVIP-PED	12,025	3463	34,135	8302	1280 × 1024	Visible	Night	2.74
TJU-PED -Traffic	13,858	2136	27,650	5244	1624 × 1200	Visible	Day, Night	2.06
TJU-PED -Campus	39,727	5204	234,455	36,161	640 × 480 ∼ 5248 × 3936	Visible	Day, Night	6.02
TJU-PED	53,585	7340	262,105	41,405	640 × 480 ∼ 5248 × 3936	Visible	Day, Night	4.98
HRBUST- LLPED (ours)	3558	711	26,774	5374	720 × 576	**Low-light**	Night	**7.53**

**Table 2:** Experimental results of wearable low-light pedestrian detection models and original YOLO models (marked with “-o”) for training and testing using the HRBUST-LLPED Dataset.

Method	Input Resolution	Train to LLPED, Test on LLPED				Infer Time (ms)
		P (%)	R (%)	AP50 (%)	AP (%)	
YOLOv5s-o	640 × 640	**93.05**	84.17	92.91	59.04	3.5
YOLOv8s-o	640 × 640	92.41	83.12	92.35	61.82	4.3
YOLOv5n-o	640 × 640	90.98	80.67	90.57	55.88	2.9
YOLOv8n-o	640 × 640	91.82	80.01	89.89	59.20	2.4
YOLOv5s	640 × 640	92.72	**85.83**	**93.93**	61.49	3.1
YOLOv8s	640 × 640	92.20	85.59	93.44	**63.64**	3.4
YOLOv5n	640 × 640	92.19	82.58	91.86	57.91	2.6
YOLOv8n	640 × 640	92.01	82.32	91.50	60.99	**1.6**

**Table 3:** Experimental results of wearable low-light pedestrian detection models trained on the LLVIP-PED Dataset and transfered to the HRBUST-LLPED Dataset.

Method	Input Resolution	Trained and Tested on LLVIP-PED Dataset				Transfered to and Tested on HRBUST-LLPED Dataset				Infer Time (ms)
		P (%)	R (%)	AP50 (%)	AP (%)	P (%)	R (%)	AP50 (%)	AP (%)	
YOLOv5s-o	640 × 640	90.35	80.60	88.59	48.93	93.76	87.35	95.61	65.22	3.5
	1280 × 1280	88.63	81.84	89.09	50.27	92.79	88.33	95.51	65.19	3.6
YOLOv8s-o	640 × 640	85.78	82.07	87.36	50.12	92.52	90.74	95.76	68.44	4.3
	1280 × 1280	91.63	80.08	88.22	50.44	93.04	90.29	95.83	68.59	15.4
YOLOv5n-o	640 × 640	92.36	79.23	88.04	47.89	92.82	86.42	94.41	61.63	2.9
	1280 × 1280	85.12	83.36	87.90	48.63	93.28	87.40	95.04	63.58	2.9
YOLOv8n-o	640 × 640	90.74	79.33	88.15	50.17	92.57	89.77	95.57	67.85	2.1
	1280 × 1280	90.95	80.39	88.66	51.57	92.20	89.69	95.42	67.18	7.3
YOLOv5s	640 × 640	**93.30**	**84.05**	**91.14**	51.05	93.62	89.83	95.74	67.27	3.2
	1280 × 1280	91.47	83.91	90.66	**52.31**	**94.19**	89.00	95.68	66.98	3.2
YOLOv8s	640 × 640	89.62	79.24	87.37	49.96	92.67	90.62	95.96	69.29	3.5
	1280 × 1280	91.58	82.55	89.54	52.29	92.43	**91.50**	**96.01**	**69.50**	12.6
YOLOv5n	640 × 640	93.27	77.58	87.84	49.55	93.75	86.02	94.56	64.34	2.6
	1280 × 1280	89.54	82.16	89.10	50.64	93.38	87.12	94.70	64.27	2.7
YOLOv8n	640 × 640	89.72	82.09	89.16	51.68	92.61	89.43	95.56	67.69	**1.7**
	1280 × 1280	90.97	82.60	89.68	52.04	92.30	89.32	95.44	67.51	5.6

**Table 4:** Experimental results of wearable low-light pedestrian detection models trained on the KITTK-PED Dataset and transferred to the HRBUST-LLPED Dataset.

Method	Input Resolution	Trained and Tested on KITTI-PED Dataset				Transfered to and Tested on HRBUST-LLPED Dataset				Infer Time (ms)
		P (%)	R (%)	AP50 (%)	AP (%)	P (%)	R (%)	AP50 (%)	AP (%)	
YOLOv5s	640 × 640	93.91	83.72	92.85	62.56	**93.80**	89.17	95.74	67.51	3.2
	1280 × 1280	**99.04**	91.16	97.29	75.69	93.16	89.41	95.77	67.58	3.2
YOLOv8s	640 × 640	94.61	88.43	95.12	74.85	93.30	91.05	**96.30**	**69.73**	3.4
	1280 × 1280	98.07	**93.34**	**97.76**	**82.85**	92.73	**91.38**	96.15	69.57	12.6
YOLOv5n	640 × 640	79.22	68.46	78.57	39.98	93.14	86.90	94.76	64.12	2.6
	1280 × 1280	96.59	85.82	94.87	66.18	92.53	87.37	94.71	64.17	2.6
YOLOv8n	640 × 640	92.30	79.69	90.93	66.31	92.49	89.36	95.44	67.90	**1.7**
	1280 × 1280	92.59	90.30	96.34	77.03	92.45	89.21	95.49	68.21	5.6

**Table 5:** Experimental results of wearable low-light pedestrian detection models trained on the KAIST-PED Dataset and transfered to the HRBUST-LLPED Dataset.

Method	Input Resolution	Trained and Tested on KAIST-PED Dataset				Transfered to and Tested on HRBUST-LLPED Dataset				Infer Time (ms)
		P (%)	R (%)	AP50 (%)	AP (%)	P (%)	R (%)	AP50 (%)	AP (%)	
YOLOv5s	640 × 640	36.09	28.22	**32.41**	13.09	**94.31**	88.74	95.74	67.42	3.2
YOLOv8s	640 × 640	**37.40**	26.50	30.41	**13.84**	93.36	**90.53**	**95.98**	**69.30**	3.4
YOLOv5n	640 × 640	37.23	**28.52**	30.06	12.58	93.21	86.92	94.50	64.04	2.6
YOLOv8n	640 × 640	36.86	26.98	29.63	12.73	92.99	88.67	95.59	68.01	**1.6**

**Table 6:** Experimental results of wearable low-light pedestrian detection models trained on the TJU-PED-Traffic Dataset and transfer to the HRBUST-LLPED Dataset.

Method	Input Resolution	Trained and Tested on TJU-PED-Traffic Dataset				Transfered to and Tested on HRBUST-LLPED Dataset				Infer Time (ms)
		P (%)	R (%)	AP50 (%)	AP (%)	P (%)	R (%)	AP50 (%)	AP (%)	
YOLOv5s	640 × 640	83.68	71.93	80.95	45.73	93.84	89.02	95.62	67.11	3.2
	1280 × 1280	88.52	77.84	87.89	53.62	**94.69**	88.97	95.88	67.75	3.2
YOLOv8s	640 × 640	84.76	73.20	82.87	48.43	92.65	90.97	96.08	69.56	3.4
	1280 × 1280	**86.84**	**81.00**	**89.30**	**56.44**	92.55	**91.11**	**96.28**	**69.30**	12.7
YOLOv5n	640 × 640	83.53	64.12	74.85	39.67	94.43	86.19	94.81	64.25	2.6
	1280 × 1280	85.91	77.64	86.26	50.62	92.91	87.55	94.91	64.70	2.6
YOLOv8n	640 × 640	83.51	67.82	78.27	44.28	92.65	89.71	95.67	67.95	**1.7**
	1280 × 1280	85.34	80.01	87.68	53.78	92.50	89.49	95.60	68.17	5.6

**Table 7:** Experimental results of wearable low-light pedestrian detection models trained on the TJU-PED-Campus Dataset and transfered to the HRBUST-LLPED Dataset.

Method	Input Resolution	Trained and Tested on TJU-PED-Campus Dataset				Transfered to and Tested on HRBUST-LLPED Dataset				Infer Time (ms)
		P (%)	R (%)	AP50 (%)	AP (%)	P (%)	R (%)	AP50 (%)	AP (%)	
YOLOv5s	640 × 640	84.95	63.04	72.23	46.55	**94.60**	89.17	96.16	68.83	3.2
	1280 × 1280	89.57	73.77	84.36	57.33	93.91	90.43	96.27	69.19	3.2
YOLOv8s	640 × 640	88.13	64.71	74.39	51.02	93.35	90.64	96.17	69.73	3.4
	1280 × 1280	**90.39**	**76.00**	**85.43**	**61.15**	92.96	**91.66**	**96.34**	**69.87**	12.6
YOLOv5n	640 × 640	85.31	57.11	66.84	40.91	93.69	88.18	95.40	65.87	2.6
	1280 × 1280	87.40	69.97	80.25	51.93	93.91	87.35	95.24	66.01	2.6
YOLOv8n	640 × 640	85.97	59.92	69.49	46.07	93.06	89.82	95.73	68.77	**1.7**
	1280 × 1280	89.68	71.49	81.80	57.21	92.89	90.19	96.00	68.71	5.6

**Table 8:** Experimental results of wearable low-light pedestrian detection models trained on the TJU-PED Dataset and transfered to the HRBUST-LLPED Dataset.

Method	Input Resolution	Trained and Tested on TJU-PED Dataset				Transfered to and Tested on HRBUST-LLPED Dataset				Infer Time (ms)
		P (%)	R (%)	AP50 (%)	AP (%)	P (%)	R (%)	AP50 (%)	AP (%)	
YOLOv5s	640 × 640	82.89	65.00	73.61	46.79	**95.15**	88.72	96.03	68.83	3.2
	1280 × 1280	89.20	74.13	84.87	57.05	94.56	89.19	96.06	68.97	3.2
YOLOv8s	640 × 640	87.27	66.56	75.88	51.04	93.46	90.66	96.24	69.59	3.4
	1280 × 1280	**90.02**	**76.89**	**86.09**	**60.67**	93.25	**91.27**	**96.29**	**69.90**	12.6
YOLOv5n	640 × 640	83.41	58.75	68.14	40.94	92.96	87.81	95.26	65.69	2.6
	1280 × 1280	87.24	70.49	80.96	51.84	93.19	88.68	95.36	65.81	2.6
YOLOv8n	640 × 640	85.35	60.88	70.80	45.82	92.67	90.27	95.75	68.53	**1.6**
	1280 × 1280	89.28	72.22	82.51	56.82	93.09	90.52	96.04	68.74	5.6

**Table 9:** The missing rate (MR), false detection rate (FDR), and incorrect localization rate (ILR) of different models on the HRBUST-LLPED Dataset with the confidence threshold set to 0.3.

Method	Input Resolution	MR (%)	FDR (%)	ILR (%)
YOLOv5s	640 × 640	6.77	10.04	2.71
	1280 × 1280	**6.66**	9.53	2.66
YOLOv8s	640 × 640	7.66	9.27	2.92
	1280 × 1280	6.98	8.72	**2.51**
YOLOv5n	640 × 640	9.03	10.22	3.62
	1280 × 1280	8.66	10.49	3.15
YOLOv8n	640 × 640	8.13	9.03	2.92
	1280 × 1280	7.88	**8.67**	2.65

## Data Availability

The datasets generated and/or analyzed during the current study are available from the corresponding author upon reasonable request.
